# Patient age affects sex-based differences in post-traumatic mortality: a national trauma registry study in Japan

**DOI:** 10.1007/s00068-021-01840-8

**Published:** 2021-12-03

**Authors:** Yutaka Umemura, Yusuke Katayama, Tetsuhisa Kitamura, Kosuke Kiyohara, Tomoya Hirose, Takeyuki Kiguchi, Jotaro Tachino, Shunichiro Nakao, Yuko Nakagawa, Takeshi Shimazu

**Affiliations:** 1 Department of Traumatology and Acute Critical Medicine, Osaka University Graduate School of Medicine, 2‑15 Yamada‑oka, Suita, Osaka 565-0871 Japan; 2 Department of Emergency and Critical Care, Osaka General Medical Center, 3‑1‑56 Bandai‑Higashi, Sumiyoshi‑ku, Osaka, Japan; 3 Division of Environmental Medicine and Population Sciences, Department of Social and Environmental Medicine, Osaka University Graduate School of Medicine, 2‑15 Yamada‑oka, Suita, Japan; 4 Department of Food Science, Faculty of Home Economics, Otsuma Women’s University Tokyo, 12 Sanban‑cho, Chiyoda‑ku, Tokyo, Japan; 5 Emergency and Critical Care Center, Osaka Police Hospital, 10‑31 Kitayama‑cho, Tennoji‑ku, Osaka, Japan; 6 Kyoto University Health Services, Yoshida‑honmachi, Sakyo‑ku, Kyoto, Japan

**Keywords:** Age factors, Mortality, Registries, Sex, Trauma

## Abstract

**Purpose:**

Sex-based differences in post-traumatic mortality have been widely discussed for quite some time. We hypothesized that age-related pathophysiologic changes would affect sex-based differences in post-traumatic mortality and aimed to verify the hypothesis using a nationwide trauma registry in Japan.

**Methods:**

This was a retrospective analysis of trauma patients registered in The Japanese Trauma Data Bank. We stratified the study population into the following three subsets based on age: (1) pediatric subset (age ≤ 14), (2) adult subset (age 15–50) and (3) senior adult subset (age ≥ 51). We evaluated both sex-based differences in mortality in each subset separately using multivariate logistic regression analysis and the two-way interaction effect for predicted survival between the continuous increase of age and the sexes using a nonlinear multivariate regression model.

**Results:**

We included 122,819 trauma patients who fulfilled the inclusion criteria and classified them into the 3 subsets according to age. Male patients were more likely to die compared to female patients only in the senior adult subset (adjusted odds ratio: 1.26; 95% confidence interval: 1.18–1.36), whereas there were no statistically significant differences in the other two subsets. Furthermore, non-linear logistic regression analysis revealed that predicted survival probability in male patients decreased more sharply in accordance with the increase of age compared to that in female patients (*p* for interaction: 0.051).

**Conclusion:**

Age-related change in post-traumatic mortality was significantly different between males and females, and male patients had a relatively higher risk of death in the older population.

## Introduction

Despite the advances made in medical technology over the past few decades, trauma remains a major cause of death worldwide. In the United States, more than 2.8 million patients with trauma were reported to be hospitalized each year, and approximately 180,000 patients died [1, 2]. Recently, a number of basic and clinical studies evaluated sex-based differences in post-traumatic morbidities and mortality with respect to immunological and pathophysiological mechanisms [3–8]. However, sex-based differences in mortality in trauma patients still remain a matter of dispute due to controversial evidence across various studies. Several studies reported that female patients were less likely to die because of their lower risk of post-traumatic infections and multiple organ dysfunction [9–12], whereas other studies reported that there was no significant difference in the risk of post-traumatic death between male and female patients [13–16]. One possible explanation for the heterogeneous evidence on sex-based differences in post-traumatic mortality was attributable to the age-related physiological, immunological and pathophysiological differences between males and females. Age-related changes in endocrine function are completely different between males and females [17]. In females, the decline of reproductive function is associated with the loss of gonadal functions, which occurs as an on/off phenomenon at the time of menopause. In males, however, reproductive function progressively declines in accordance with the decrease of testicular function [18]. Various studies reported that the favorable outcomes seen in female patients with severe trauma were associated with the sex hormones [19–21], and therefore it would be reasonable to assume that sex-based differences in post-traumatic mortality would be different between the age classes due to sex differences in aging related to the decline of sex hormones.

We thus hypothesized that age-related pathophysiologic changes would affect sex-based differences in post-traumatic mortality in certain age classes. This study aimed to evaluate the detailed association between age and sex-based difference in outcomes of patients with severe trauma using a nationwide trauma registry in Japan.

## Materials and methods

### Study population

This is a retrospective analysis of trauma patients registered in The Japanese Trauma Data Bank (JTDB), which is a nationwide trauma registry database managed by The Japanese Association for the Surgery of Trauma. Data registration in the JTDB was launched in 2003, and approximately 290,000 patients with trauma and burn were enrolled by 2017 [22]. Among them, we included all trauma patients with an Injury Severity Score (ISS) of 16 or more at the time of hospital arrival. We excluded patients who were pregnant or in cardiopulmonary arrest at the time of hospital arrival. This study followed the principles of the Declaration of Helsinki and was approved by the institutional review board of Osaka University Hospital (No. 16260). Because of the anonymous and retrospective nature of this study and the personnel identifiers were removed beforehand from the JTDB database, the patients’ right to informed consent was waived.

### Data collection

The JTDB was compiled via the Internet by the investigators of 256 major emergency medical institutions across Japan, which were regarded as being equal to Level I trauma centers in the United States. Patients were followed up until hospital discharge or death during their hospitalization. A case report form was developed for the study, and the following information was obtained: age, sex, mechanism of injury, onset-to-arrival time, pre-existing comorbidities, Abbreviated Injury Scale (AIS) code (version 1998), ISS, Revised Trauma Score (RTS), vital signs on arrival, medical treatment including interventional radiology and surgical operations, and outcomes. ISS was calculated from the top three scores of the AIS at nine sites classified by the AIS code. The primary outcome measure was all-cause in-hospital mortality. As secondary outcomes, we recorded several post-traumatic complications, such as respiratory failure, circulatory failure, neuropsychiatric disorders, gastrointestinal failure, coagulopathy, osteopathy, pneumonia/pyothorax and other infectious diseases. We provide detailed information about the actual diagnoses included in these complication categories in Table 1.

**Table 1:** Categorization of post-traumatic complications in this study

Respiratory failure	Pulmonary edema, Atelectasis, Pulmonary embolism, Acute respiratory distress syndrome
Circulatory failure	Acute myocardial infarction, Arrhythmia, Prolonged shock, Cardiopulmonary arrest, Abdominal compartment syndrome
Neuropsychiatric disorders	Diabetes insipidus, Hydrocephalus, Fat embolism, Liquorrhea, Higher brain dysfunction, Post‐traumatic stress disorder
Gastrointestinal failure	Stress ulcer, Ileus, Acute pancreatitis, Acute liver failure
Coagulopathy	Disseminated intravascular coagulation, Thrombocytopenia
Osteopathy	Compartment syndrome, Re-fracture, False joint
Pneumonia/pyothorax	Pneumonia, Pyothorax
Other infectious diseases	Bacteremia, Sepsis, Abdominal abscess, Urinary tract infection, Infectious enteritis, Wound infection, Wound dehiscence, Bedsore, Hypothermia, Cholecystitis, Osteomyelitis, Meningitis

### Statistical analysis

The aim of this study was to determine whether the sex-based differences in post-traumatic mortality were different according to patient age classes and to explore an age class in which the risk of trauma death was different between males and females. We thus divided the study population according to the ages at which the sex hormones in females suddenly and dramatically change and stratified it into three subsets based on the following ages in years: (1) pediatric subset (age ≤ 14), (2) adult subset (age 15–50), and (3) senior adult subset (age ≥ 51). We evaluated sex-based differences in the primary and secondary outcomes in each subset separately using multivariate logistic regression analysis adjusted by 32 relevant covariates including age, mechanism of injury, onset-to-arrival time, pre-existing comorbidities, injury severity scores, vital signs on arrival at hospital and therapeutic interventions. Detailed combinations of the covariates are listed in Table 2.

**Table 2:** The 32 variables used to adjust the logistic regression models

Patient characteristics	(1) Age, (2) Sex
Vital signs	(3) Systolic blood pressure, (4) Diastolic blood pressure, (5) Respiratory rate, (6) Heart rate, (7) Glasgow Coma Scale
Mechanism of injury	(8) Traffic accident, (9) Fall injury, (10) Penetrating injury
Pre-existing condition	(11) Cardiovascular diseases, (12) Respiratory failure, (13) Gastrointestinal diseases, (14) Metabolic diseases, (15) Neuropsychiatric disorders, (16) Immunodeficiency, (17) Cancer, (18) Chronic hemodialysis
Abbreviated Injury Scale	(19) Head, (20) Face, (21) Neck, (22) Thorax, (23) Abdomen, (24) Spine, (25) Upper extremity, (26) Lower extremity including pelvis
Operation	(27) Head, (28) Chest, (29) Abdomen, (30) Trans-arterial embolization, (31) Limb, (32) Other site

For the primary outcome of in-hospital mortality, we evaluated the detailed association between the continuous increase of age and sex-based differences in mortality using a nonlinear regression model. In these analyses, we evaluated whether the age-related changes in mortality were statistically different between male and female patients by calculating the two-way interaction effects between sex, mortality and the increase of age as the continuous variable. Assessments of the interaction effects were conducted with multivariate logistic regression analysis models including the same covariates described in Table 2 and a two-way interaction term. We also evaluated the continuous associations between age and sex-based differences in mortality in several patient subgroups according to the mechanism of injury (blunt or penetrating trauma) and the presence of severe head trauma (AIS ≥ 3).

Baseline characteristics were compared by the Student *t*-test or chi-square test. Descriptive statistics are summarized as group means with the standard error for the continuous variables and frequencies with percentages for the categorical variables. All statistical inferences without interaction effects were two-sided, and a *p* value of < 0.05 indicated statistical significance. Because of the underpowered nature of the interaction analysis, we used a two-sided significance level of 0.20 with statistical inferences for the interaction effects [23]. All statistical analyses were conducted using STATA Data Analysis and Statistical Software version 15.0 (StataCorp, College Station, TX).

## Results

### Study population

**Fig. 1:**
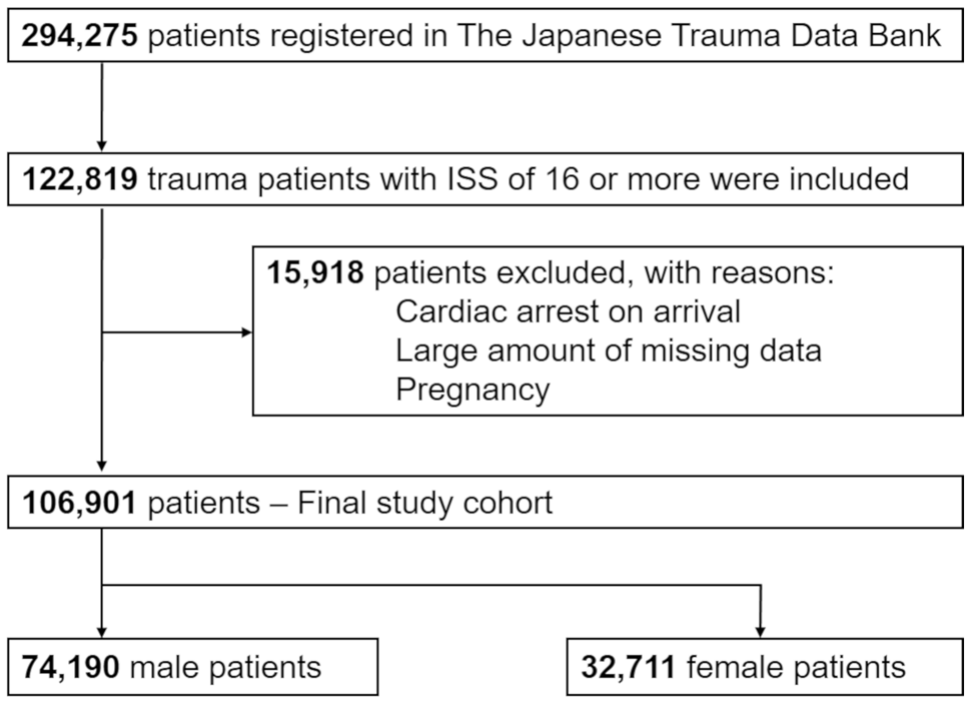
Patient flow diagram. *ISS* Injury Severity Score

The patient flow diagram is shown in Fig. 1. Among 294,275 patients registered in the JTDB by 2017, we included 122,819 trauma patients with an ISS of 16 or more at the time of hospital arrival. After excluding 15,918 patients who were pregnant, in cardiopulmonary arrest on arrival or missing valuable data, we analyzed 106,901 patients as the final study cohort. The male group comprised 74,190 patients, and the female group comprised 32,711 patients.

Baseline characteristics, vital signs, severity scores, post-traumatic complications and in-hospital mortality ratio of the male and female patients in each subset based on age are shown in Table 3. Although male groups had a higher mean age in the pediatric and adult subsets, the female group had a significantly higher mean age in the senior adult subset. Distribution of the mechanisms of injury was different between male and female patients in the adult and senior adult subsets, i.e., traffic accident was the most common cause in males of the adult subset, whereas fall was the most common cause in males of the senior adult subset. Differences of pre-existing comorbidities tended to be apparent in the older subsets. In the senior adult subset, male patients had significantly higher rates of respiratory, gastrointestinal and metabolic diseases and cancer but significantly lower rates of cardiovascular, neuropsychiatric and immunodeficiency diseases. Several vital signs and severity scores were statistically significantly different between male and female patients, especially in the adult and senior adult subsets; however, many of these differences were clinically very slight. Male patients in the senior adult subset had significantly higher ratios of many post-traumatic complications such as respiratory failure, neuropsychiatric disorders, pneumonia/pyothorax and other infectious diseases. There were no significant differences in crude mortality ratio between the male and female patients in all three subsets (3.1 vs 3.6%, 8.7 vs 8.6% and 14.8 vs 14.8%, respectively).

**Table 3:** Baseline characteristics and severity of injury in each age class

	Age < 15			Age:15–50			Age > 50		
	Female	Male	*p*	Female	Male	*p*	Female	Male	*p*
	*n* = 1687	*n* = 3626		*n* = 7597	*n* = 25,070		*n* = 22,759	*n* = 43,963	
Age (years)	6.8 ± 0.1	7.4 ± 0.1	< 0.001	31.6 ± 0.1	32.4 ± 0.1	< 0.001	74 ± 0.1	69.5 ± 0.1	< 0.001
Mechanism of injury									
Traffic accident	852 (50.5%)	1834 (50.6%)	0.18	3969 (52.2%)	14,824 (59.1%)	< 0.001	10,160 (44.6%)	15,149 (34.5%)	< 0.001
Fall	628 (37.2%)	1286 (35.5%)		2833 (37.3%)	6458 (25.8%)		11,077 (48.7%)	24,326 (55.3%)	
Other	207 (12.3%)	506 (14%)		795 (10.5%)	3788 (15.1%)		1522 (6.7%)	4488 (10.2%)	
Pre-existing conditions									
Cardiovascular	14 (0.8%)	31 (0.9%)	0.926	159 (2.1%)	942 (3.8%)	< 0.001	8584 (37.7%)	15,188 (34.6%)	< 0.001
Respiratory	76 (4.5%)	243 (6.7%)	0.002	250 (3.3%)	988 (3.9%)	0.009	750 (3.3%)	1640 (3.7%)	0.004
Gastrointestinal	10 (0.6%)	18 (0.5%)	0.652	201 (2.7%)	859 (3.4%)	0.001	1753 (7.7%)	4497 (10.2%)	< 0.001
Metabolic	7 (0.4%)	10 (0.3%)	0.403	138 (1.8%)	712 (2.8%)	< 0.001	3451 (15.2%)	7456 (17%)	< 0.001
Neuropsychiatric	57 (3.4%)	111 (3.1%)	0.538	1956 (25.8%)	2004 (8%)	< 0.001	4557 (20%)	6439 (14.7%)	< 0.001
Immunodeficiency	43 (2.6%)	101 (2.8%)	0.621	242 (3.2%)	581 (2.3%)	< 0.001	1472 (6.5%)	2258 (5.1%)	< 0.001
Cancer	0 (0%)	1 (0%)	0.495	31 (0.4%)	29 (0.1%)	< 0.001	530 (2.3%)	1394 (3.2%)	< 0.001
Hemodialysis	0 (0%)	0 (0%)	N.A	7 (0.1%)	29 (0.1%)	0.588	413 (1.8%)	858 (2%)	0.22
sBP (mmHg)	113.3 ± 0.6	117.8 ± 0.4	< 0.001	115.3 ± 0.3	127.2 ± 0.2	< 0.001	140.1 ± 0.3	139.4 ± 0.2	0.026
RR (/min)	25.8 ± 0.2	25.2 ± 0.2	0.041	23.2 ± 0.1	23.0 ± 0.1	0.136	21.5 ± 0.0	21.4 ± 0.0	0.252
HR (/min)	114.5 ± 0.8	107.9 ± 0.5	< 0.001	94.2 ± 0.3	91.7 ± 0.2	< 0.001	85.4 ± 0.1	84.6 ± 0.1	< 0.001
GCS	12.4 ± 0.1	12.3 ± 0.1	0.566	12.0 ± 0.0	12.2 ± 0.0	< 0.001	12.2 ± 0.0	12.3 ± 0.0	0.01
RTS	7.1 ± 0.0	7.1 ± 0.0	0.487	7 ± 0.0	7.1 ± 0.0	< 0.001	7.1 ± 0.0	7.1 ± 0.0	0.31
ISS	22.5 ± 0.2	22.3 ± 0.1	0.379	26.3 ± 0.1	25.3 ± 0.1	< 0.001	23.8 ± 0.1	23.4 ± 0.0	< 0.001
Post-traumatic complications									
Respiratory failure	24 (1.4%)	52 (1.4%)	0.974	214 (2.8%)	809 (3.2%)	0.072	689 (3%)	1525 (3.5%)	0.002
Circulatory failure	23 (1.4%)	30 (0.8%)	0.067	207 (2.7%)	605 (2.4%)	0.127	895 (3.9%)	1642 (3.7%)	0.209
Neuropsychiatric disorders	84 (5%)	165 (4.6%)	0.491	517 (6.8%)	1685 (6.7%)	0.798	1154 (5.1%)	2728 (6.2%)	< 0.001
Pneumonia/pyothorax	26 (1.5%)	46 (1.3%)	0.424	184 (2.4%)	840 (3.4%)	< 0.001	758 (3.3%)	2542 (5.8%)	< 0.001
Other infectious diseases	43 (2.5%)	41 (1.1%)	< 0.001	380 (5%)	1253 (5%)	0.989	1157 (5.1%)	2543 (5.8%)	< 0.001
In-hospital mortality	53 (3.1%)	132 (3.6%)	0.356	663 (8.7%)	2148 (8.6%)	0.665	3366 (14.8%)	6477 (14.8%)	0.858

*GCS* indicates Glasgow Coma Scale, *HR* heart rate, *ISS* Injury Severity Score, *RR* respiratory rate, *RTS* Revised Trauma Score, *sBP* systolic blood pressure

### Sex-based differences in three subsets

**Fig. 2:**
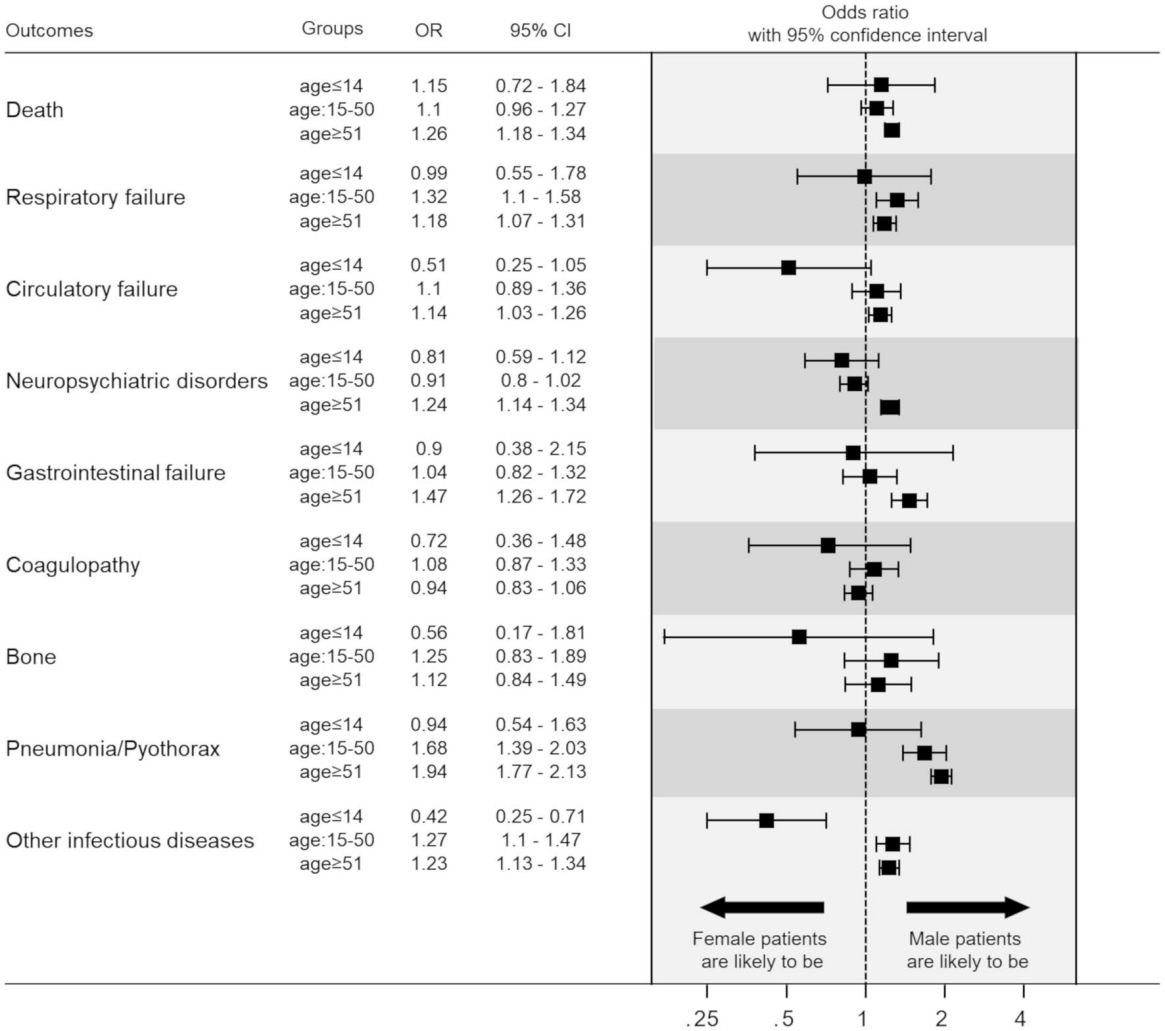
Summary of sex-based differences in post-traumatic survival and complications in the three age classes

The results of multivariate logistic regression analyses for primary and secondary outcomes after adjustment by the covariates are shown in Fig. 2. The sex-based difference in the risk of trauma death was statistically significant only in the senior adult subset, i.e., male patients had a significantly higher risk of death compared to female patients (adjusted odds ratio: 1.26; 95% confidence interval: 1.18–1.36). In contrast, there were no sex-based differences in the risk of trauma death in the pediatric and adult subsets. Similarly, male patients had statistically significantly higher risks of circulatory failure, neuropsychiatric disorders and gastrointestinal failure only in the senior adult subset and had higher risks of respiratory failure, pneumonia/pyothorax and other infectious diseases in the adult and senior adult subsets. We observed no statistically significant sex-based differences in any of the secondary outcomes in the pediatric subset.

### Sex-based differences in age-related change in mortality

**Fig. 3:**
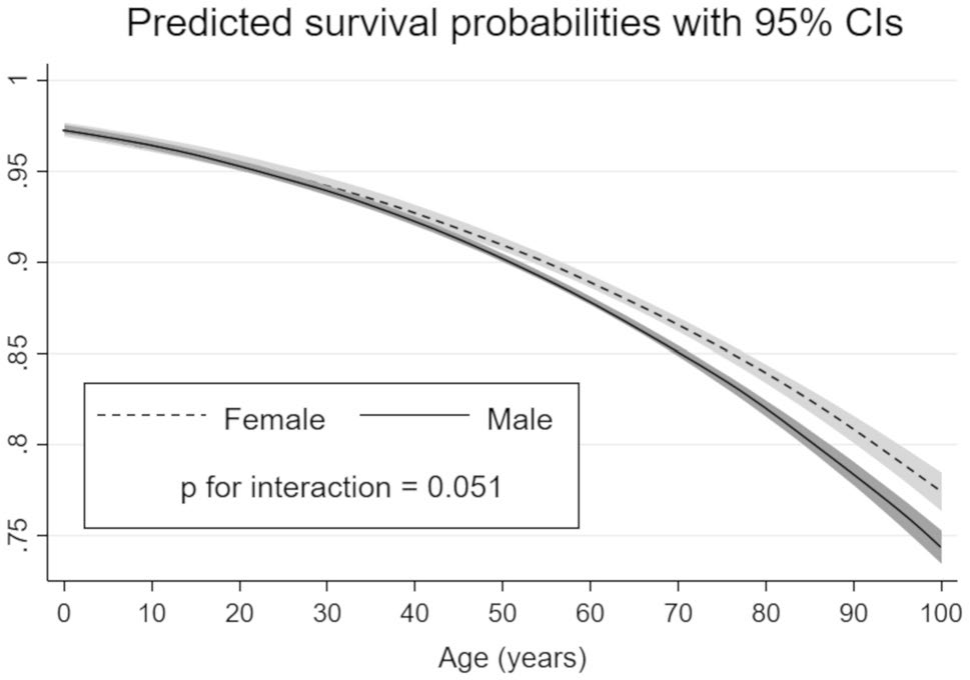
Age-related change of predicted survival probabilities in male and female patients. The solid black line represents the estimated mortality in the male patients, and the dashed black line represents the estimated mortality in the female patients

The results of a non-linear multivariate logistic regression analysis that included a three-way interaction term between sex, mortality and the increase of age as the continuous variable to examine how the sex-based difference in mortality changed in accordance with the increase of age are shown in Fig. 3. In the younger-aged patients, the predicted survival probabilities after trauma in both sexes were similarly decreased in accordance with their increase of age. However, as age increased over approximately 50 years old, the predicted survival probabilities in the male group decreased more sharply compared to those in the female group, and the interaction effects were statistically significant (*p* = 0.051). In other words, age-related change in post-traumatic mortality was significantly different between the males and females, and male patients had a higher risk of post-traumatic death in the older population.

### Subgroup analyses

**Fig. 4:**
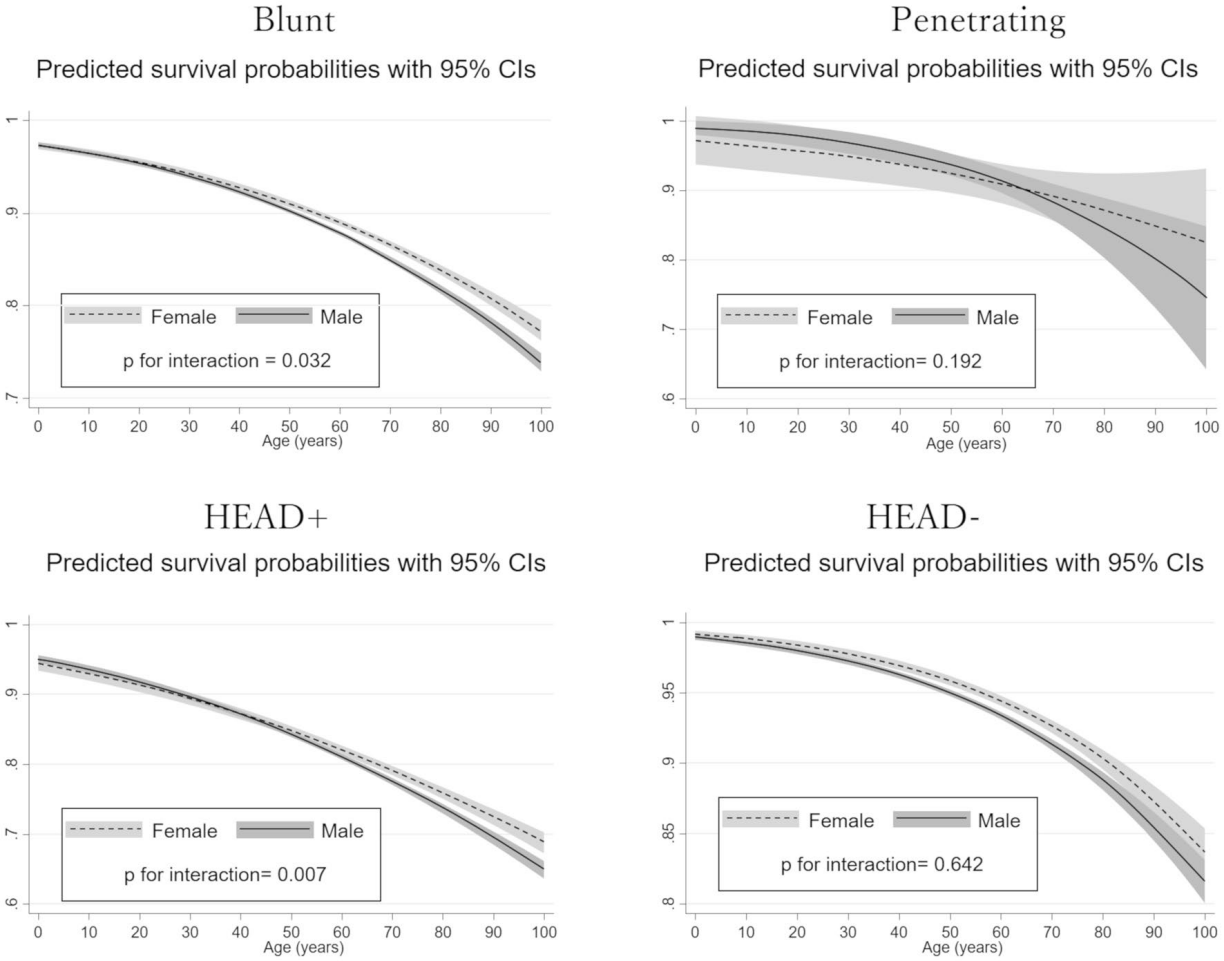
Age-related change of predicted survival probabilities between the sexes in several subgroups. The solid black lines represent the estimated mortality in the male patients, and the dashed black lines represent the estimated mortality in the female patients

The results of non-linear multivariate logistic regression analyses in several subgroups are shown in Fig. 4. In the blunt trauma patients, the predicted survival probabilities in the male group sharply decreased as age increased over approximately 50 years old, and we observed a strong interaction effect between the age-related decrease of mortality and sex (*p* for interaction = 0.032). However, in the patients with penetrating trauma, the difference in predicted survival probabilities between the two sexes was small, and there was only a weak interaction effect (*p* = 0.192). Similarly, a significant interaction between age-related decrease of mortality and sex was observed only in patients with severe head trauma (*p* for interaction = 0.002), but no such significant interaction was observed in patients without severe head trauma (*p* for interaction = 0.257).

## Discussion

A number of studies so far have evaluated differences in the clinical course of trauma between male and female patients, but no definite evidence on sex-based differences in post-traumatic mortality has been reported. The present study using a nationwide trauma registry in Japan represented an attempt to evaluate the association between aging and the sex-based differences in post-traumatic mortality. It provided clear evidence that the sex-based differences in post-traumatic mortality fluctuate according to patient age classes, and male patients were more likely to die compared to females only in the senior adult population. Further, non-linear logistic regression analysis revealed that the age-related increase in predicted mortality was significantly more serious in male patients compared to that in female patients. Interestingly, this association was especially remarkable in specific subgroups such as patients suffering blunt trauma or severe head trauma.

### Sex-based difference in aging

Multiple lines of evidence have shown that females have a survival advantage in various life-threatening diseases such as sepsis, heart failure, out-of-hospital cardiac arrest and trauma [24–28]. Similarly, the present study showed that females were more likely to survive after severe trauma, but the survival benefit of females was found only in the senior adult population. One possible reason for this age-related difference in the survival advantage of females was partly attributable to the aging process, which is qualitatively different between the sexes. Aging is associated with a decrease in the physiological function of the endocrine system, and the secretion of sex hormones declines in accordance with advancing age [17]. The aging-related decline of sex hormones is quite different between the sexes [29] and is reported to play an important role in the determination of sexual dimorphism in the decline of the immune system, namely, immunosenescence [30]. Immunosenescence refers to the gradual deterioration of immune responses in accordance with advancing age, and it increases morbidity and mortality due to infections. Several studies have suggested that the progression to immunosenescence associated with the decline of sex hormones is faster in males than in females [31]. Actually, in the present study, male patients had a higher risk of post-traumatic infectious complications such as pneumonia and pyothorax especially in the older populations, also suggesting that immunosenescence was faster in male patients. Similarly, the incidence rates of respiratory failure and neuropsychiatric disorders were significantly higher only in the older male patients, potentially leading to the higher rate of death in this population. A combination of genetic, environmental and social factors and immunological differences could contribute to the lower risk of post-traumatic death in females in older populations.

### Agreement and disagreement with past studies

So far, several studies have evaluated age-related variation in sex-based differences in terms of post-traumatic risk of death. Zhu et al. reported that the survival advantage of female trauma patients was different between age classes and was particularly notable in the patients younger than 45 years [32]. Additionally, Haider et al. reported that mortality was significantly lower in adolescent girls than boys with traumatic shock, but there was no significant sex-based difference in mortality in prepubescent children [33].

Our study agreed with these observational studies regarding the survival advantage of female trauma patients seen only in the specific age classes, but it disagreed with them regarding the age categories in which the sex-based difference in post-traumatic mortality was most significantly observed, i.e., the previous studies showed a difference in sex-based survival in younger populations, whereas our findings showed it in an older population. There are several possible explanations for these divergent results. First, the present study included a nationwide and extremely large sample size, which reduced type 2 error and enhanced the precision of the study results. Second, to provide robust evidence on sex-based differences in the risk of post-traumatic death, multivariate regression analysis adjusted for the imbalances in injury severity and baseline characteristics was conducted in the present study. Actually, there was no significant difference in the non-adjusted crude mortality ratio between male and female patients in the senior adult subset, which agreed with the previous studies. Third, the definition of cut-off age was different from that of the previous studies and thus could also be responsible for the divergent results. To concur with the uncertainty of the results caused by the definition of age classes, we also conducted non-linear multivariate logistic regression analysis to examine how the sex-based difference in mortality changed in accordance with the continuous increase of age. This analysis visually provided novel and comprehensible evidence about the impact of patient age on sex-based differences in post-traumatic mortality.

### Differences according to subgroups

Our subgroup analysis revealed that the sharp age-related increase in mortality in male patients was especially remarkable in the patients with blunt trauma and severe head trauma. In the head trauma subgroup, our findings might be attributable to the higher incidence of infectious complications. Infectious diseases have been reported to frequently complicate acute traumatic brain injury and significantly worsen the prognosis of trauma patients [34, 35]. Therefore, our result showing a remarkable difference in sex-based mortality in the head trauma subgroup could be partly explained by the higher incidence of pneumonia and other infectious diseases observed in the older male population. However, we cannot offer a clear explanation for the difference between the blunt and penetrating trauma patients due to the lack of sufficient evidence on differences in age-related or sex-based pathophysiological changes between blunt and penetrating trauma. In Japan, the number of patients with severe penetrating trauma is relatively small compared to the large number suffering severe blunt trauma, partly due to Japan’s firearms regulations. We thus plan to perform further analyses to elucidate this point with high-quality evidence in the future.

### Limitations

We acknowledge several limitations in our study. First, there was an imbalance in the numbers of patients in the age classes in this study. We classified the study population according to the general age at which sex hormones in females suddenly and dramatically change and made no attempt to create subsets with equal populations. Therefore, we cannot deny the risk of bias in the statistical precision (confidence interval) of the measurements, especially in the pediatric subset. Therefore, further research containing an extra-large scale pediatric population is required to provide definite evidence about sex-based differences in post-traumatic mortality in this population. Second, the injury severity scores and baseline characteristics were different between the sexes. To cope with these imbalances associated with an observational design, we conducted multivariate analysis. However, it is hard to completely remove the effects of observed confounders because multiple unmeasured variables may account for the differences in outcome. Third, the present dataset did not include laboratory, radiological or physiological data that could explain the pathophysiological mechanisms of sex-based differences in host response to trauma. We considered the age-related change in the endocrine systems, proved by multiple lines of evidence, to be a key pathology; however, further investigation will be needed to reveal the detailed pathophysiological mechanisms.

## Conclusion

The present study using a nationwide trauma registry in Japan suggested that the sex-based differences in post-traumatic mortality differed according to patient age, and female patients were more likely to survive only in the older population. The sex-based difference in age-related pathophysiologic changes is a key factor in the prediction of post-traumatic survival.

## Data Availability

The data that support the findings of this study are available from the JTDB, but the availability of these data is restricted.
